# Support needs of patients with obesity in primary care: a practice-list survey

**DOI:** 10.1186/s12875-017-0703-4

**Published:** 2018-01-08

**Authors:** Elizabeth H. Evans, Kirby Sainsbury, Dominika Kwasnicka, Alex Bolster, Vera Araujo-Soares, Falko F. Sniehotta

**Affiliations:** 1 Institute of Health & Society, Newcastle University, Newcastle upon Tyne, NE2 4AX UK; 2 School of Psychology and Speech Pathology, Curtin University, GPO Box U1987, Perth, WA 6845 Australia

**Keywords:** Obesity, Overweight, Primary health care

## Abstract

**Background:**

UK guidelines recommend that patients with obesity in primary care receive opportunistic weight loss advice from health care professionals, but there is a lack of research into the characteristics and existing weight management practices of these patients. The aim of this study was to characterise primary care patients with obesity in England, to inform the screening, support, and referral options appropriate to this group.

**Methods:**

We surveyed 1309 patients registered at 15 GP practices in North East England, aged ≥18 years and with objectively recorded obesity (BMI ≥ 30 kg/m^2^). Study participants reported their weight history, health status, past and current weight loss activities, motivating factors, weight loss strategies used, professional support received, and perceived barriers to weight loss.

**Results:**

62% of participants were actively trying to lose weight, and a further 15% had attempted and discontinued weight loss in the last 12 months. Only 20% of the sample had sought GP support for weight loss in the last 12 months; instead, most efforts to lose weight were self-guided and did not use evidence-based strategies. Those who sought GP weight loss support were likely to use it and find it motivating. Participants had attempted weight loss on multiple previous occasions and overall felt less confident and successful at maintaining weight loss than losing it. Participants at greatest clinical risk (higher BMI and more health conditions) reported particularly low confidence and multiple barriers to weight loss, but were nevertheless highly motivated to lose weight and keep it off.

**Conclusions:**

We identified the need for informational, structural, and weight loss maintenance-specific support for GP patients with objectively-recorded obesity. Study participants were motivated to lose weight and keep it off, but lacked the confidence and understanding of effective strategies required to do this. GP weight loss support was acceptable and useful but underutilised, indicating that screening and brief referral interventions to structured programmes may augment patients’ current weight management activities and meet key support needs whilst optimising limited primary care resources.

**Electronic supplementary material:**

The online version of this article (10.1186/s12875-017-0703-4) contains supplementary material, which is available to authorized users.

## Background

A quarter of UK adults have a body weight that places them in the range for obesity (≥30 kg/m^2^) [1], and consequently at elevated risk for weight-related morbidity [2] and mortality [3]. Sustained weight loss of 5–10% is effective in reducing medical risk for these individuals [4]. UK guidelines therefore recommend that primary care staff screen for obesity in the office setting, and opportunistically encourage affected individuals to lose weight by offering advice and signposting to available lifestyle management services [5–7]. Despite recent evidence that brief referral interventions initiated in GP settings are effective [8, 9], concerns about the acceptability of providing weight loss advice [10] have meant that advice and referral are seldom done in practice [11].

Little is known about the potential recipients of weight loss advice: the characteristics, needs and weight management experiences of patients classified as obese presenting in primary care. Most previous research into weight loss attempts in the UK has focused upon the general population, notably via the Health Survey for England (HSE) [12, 13]. HSE participants with a BMI ≥30 kg/m^2^ were more likely to report an ongoing weight loss attempt than those with a BMI in the healthy range (76 vs. 30%), and this likelihood was higher still if weight loss had been advised by a health care professional [14]. It is not known whether weight loss attempts in the general practice population with obesity are equally prevalent. Overall, information is needed about the broader weight-related context of patients with obesity in primary care, particularly given the higher intensity of primary healthcare usage in this group [15]. Individuals with obesity are frequently subject to stigmatisation and discrimination [16, 17], and it is vital that weight management offered in primary care is non-judgemental, supportive and both informed by and sensitive to patients’ previous experiences.

In the current study, we therefore examined the weight management activities, experiences, needs and attitudes of individuals with obesity in a primary care population, using a postal survey distributed via GP surgeries. We asked participants about their weight history, the importance they placed on weight loss and weight loss maintenance, confidence, past success, motivating factors, use of weight loss strategies, the impact of health conditions on weight control, and other perceived barriers. The aim of this study was to characterise primary care patients with obesity in England, with a view to informing recommendations for the screening, support, and referral options appropriate to this previously undescribed group.

## Method

### Participants and recruitment procedure

Practice administrative staff identified potential participants at 15 General Practices in North East England (Northumberland and Tyne & Wear) by screening medical record databases in July and August 2015. Patients were eligible if they were aged ≥18 years and had a read code for BMI ≥30 kg/m^2^ or for obesity (any type of obesity coded within EMIS/SystmOne) recorded in their notes within the past 12 months. The practice sent a study pack to eligible patients consisting of a personalised letter from the practice, an information sheet and consent form, the study survey, and a postage-paid return envelope addressed to the University. As a gesture of thanks, participants were invited to enter a prize draw to win one of three £100 shopping vouchers by completing an entry form and returning it with the survey in the envelope. The North East and Cumbria Clinical Research Network facilitated recruitment of practices to the study and survey distribution. Ethics approval was obtained on 6 February 2014 from the East Midlands-Derby National Research Ethics Service (REC: 14/EM/0069).

### Materials

The survey was designed to obtain the following information from participants:

1. Demographic details, self-rated health (rated from 1 to 5, excellent to poor), and details of current health conditions;
2. Current height and weight, highest and lowest adult weights (excluding pregnancy), number of previous weight loss attempts (lifetime), whether a weight loss attempt was ongoing and, if not, the length of time since last trying to lose weight;
3. In reference to participants’ previous weight management efforts *overall,* confidence to lose weight and maintain weight loss, the perceived importance of weight loss and weight loss maintenance, and previous weight loss and weight loss maintenance success (all rated from 1 to 6, very low to very high);
4. Motivating factors for weight loss in participants’ most recent weight management efforts (participants selected from a list of 13 common motivating factors and/or specified any other factor(s));
5. Use of weight loss methods within the last 12 months (participants selected from a list of 21 common weight loss strategies and/or specified ‘any other strategy/ies), use and source of professional weight loss support within this timeframe, usual frequency of self-weighing, and current levels of physical activity (vigorous, moderate and walking), using the International Physical Activity Questionnaire - Short Form (IPAQ-SF) [18];
6. Perceived barriers to weight loss in participants’ most recent weight management efforts (participants selected from a list of 9 common barriers to weight loss and/or specified any other barrier(s)).
7. Any other information participants wished to provide, in a free-text box.

The complete set of questions is provided as an additional file (Additional file 1).

### Data analysis

Data were manually entered and analysed using SPSS version 21 [19]; a subset were second-entered to check for accuracy. Ratio and interval-level data were examined for the assumptions of parametric analysis through visual inspection of histograms and Kolmogorov-Smirnov calculations. BMI was computed from self-reported height and weight data using the following formula: BMI = weight (kg) / height^2^ (m). The *p* value threshold was set a priori at *p* < 0.001 to avoid type 1 errors as a result of multiple comparisons. Missing data were dealt with using pairwise deletion as appropriate, and non-plausible height and weight data were excluded from analyses. Pearson’s correlation coefficient was used to express associations between variables, and between-group differences were examined using independent-samples *t*-tests and Pearson chi-squared tests. Free-text data provided in the comments box were analysed using a content-analytic approach [20], in which a coding frame was developed to describe the thematic content and codes assigned to comments by two independent coders.

## Results

### Sample characteristics

Participants returned 1309 completed surveys out of 5800 sent (response rate = 23%). The sample had a mean age of 59 ± 14 years (range = 18–97) and 54% were women. The majority (90%) self-identified as white British, whilst the remainder self-identified as belonging to other categories (e.g., White European: 1%; mixed race: 1%) or preferred not to say (8%). Roughly a third of the sample was married or cohabitating (61% and 7% respectively); the remainder was single (11%), separated or divorced (10%), or widowed (8%). Just over half the sample had completed education beyond sixth form (degree: 18%; vocational: 35%); the other half had completed A-levels (6%); GCSEs (14%); other (1%); or had education below high school level (20%; prefer not to say/missing: 6%).

### Health status and health conditions

Most participants (83%) reported at least one current health condition, including hypertension (45%), chronic pain (42%), mobility problems (34%), diabetes (27%), and depression and/or anxiety (25%). Respondents who reported at least one health condition were older (*r* = 0.29, *p* < 0.001) and had a significantly higher BMI (*r* = 0.13, *p* < 0.001) than those without. Participants described their overall health as poor (12%), fair (35%), good (38%), very good (13%), or excellent (1%). Participants with higher BMIs reported poorer health (*r* = −0.17, *p* < 0.001); self-rated health did not vary with age (*r* = −0.05, *p* = 0.11) or gender (*r* = 0.01, *p* = 0.85).

### Weight and weight loss history

Table 1 summarises participants’ weight, weight history, and BMI by gender. According to current self-reported weight, 83% of the sample fell into the obese category (BMI ≥30 kg/m^2^), whilst 17% was classified as overweight (BMI 25–29.9 kg/m^2^). A BMI in the healthy range was reported by 7 participants (0.5%). Women had significantly higher current (*t*_(1243)_ = −7.5, *p* < 0.001) and lifetime highest (*t*_(1238)_ = − 7.5, *p* < 0.001) BMIs compared to men, but lower lifetime lowest BMIs than men (*t*_(1226)_ = 1.7, *p* < 0.001). Around a third (31%) of the sample reported that their weight had stayed the same (+/−2 kg) over the past 12 months; equivalent numbers reported weight loss (16%) and weight gain (16%), and 36% said their weight had fluctuated during this time.

**Table 1:** Current and lifetime highest/lowest weights and corresponding BMIs by gender

		Women (*n* = 703)				Men (*n* = 583)		
	*N*	Mean	SD	Min-max	*N*	Mean	SD	Min-max
Current weight (kg)	684	94.1	19.3	53.6–280	577	104.6	16.1	66.8–210
Current BMI (kg/m^2^)	684	35.7	6.8	20.4–91.2	570	33.3	4.6	18.5–69.4
Highest lifetime weight (kg)	684	102.3	23.8	56.4–336	573	112.7	19.7	70–230
Highest BMI (kg/m^2^)	683	38.8	8.0	21.2–92.6	566	35.8	5.8	18.8–79.4
Lowest lifetime weight (kg)	685	68.5	18.2	42.7–200	558	83.2	14.9	41.8–156
Lowest BMI (kg/m^2^)	684	26.0	6.4	16.2–72.6	551	26.5	4.6	14.1–55.5

Gender data missing for 23 participants. Highest lifetime weight excludes pregnancy

Almost two thirds (62%) of the sample reported that they were currently trying to lose weight. Among these participants, this current attempt had lasted anywhere from 1 day to more than 50 years (median = 1 year; IQR = 17–104 weeks). Only 7% of participants reported having never attempted weight loss, while for the remainder, their most recent attempt was within the last 3 months (3.2%), within the last 6 months (2.7%), within the last 12 months (9.1%), or more than 12 months ago (15.1%). A greater proportion of the women (70%) were currently attempting weight loss than men (53%; *χ*^*2*^_(2)_ = 45.7, *p* < 0.001); women also reported a greater number of weight loss attempts in their lifetimes (16 ± 34 attempts) than men (9 ± 21 attempts; *t*_(1004)_ = −3.9, *p* < 0.001). Participants currently trying to lose weight were significantly younger (57.6 ± 14.8 years) than those who were not (61.1 ± 13.4 years; *t*_(1243)_ = 4.1, *p* < 0.001).

### Weight loss: Importance, perceived success, and confidence

Table 2 shows the mean values for, and associations amongst, participants’ ratings of global importance, confidence, and perceived success for weight loss and weight loss maintenance, BMI, number of previous weight loss attempts, and number of self-reported health conditions. Overall, participants considered that weight loss and weight loss maintenance were extremely important. They perceived themselves to have been slightly successful at weight loss in the past, but unsuccessful at previous weight loss maintenance. Their confidence to lose weight was slightly low, but their confidence to keep weight off after losing it was significantly lower (*t*_*(1183)*_ = 15.33, *p* < 0.001).

**Table 2:** Importance, success, and confidence ratings, and correlations with BMI, weight loss attempts, and number of health conditions

		Correlations [99% confidence intervals]		
	Mean (SD)	Current BMI	No. WL attempts	No. health conditions
Importance: WL	5.09 (1.14)	0.19 [0.12 to 0.26]	0.12 [0.05 to 0.19]	.06 [−0.01 to 0.13]
Importance: WLM	5.15 (1.11)	0.18 [0.11 to 0.25]	0.11 [0.4 to 0.18]	.07 [0.0 to 0.14]
Success: WL	3.63 (1.40)	−0.07 [−0.14 to 0.0]	0.01 [−0.08 to 0.06]	−.08 [−0.15 to −0.01]
Success: WLM	2.43 (1.30)	−0.23 [−0.3 to −0.16]	−0.15 [−0.22 to −0.08]	−.06 [−0.13 to 0.01]
Confidence: WL	3.34 (1.43)	−0.15 [−0.22 to −0.08]	−0.05 [−0.12 to 0.02]	−.15 [−0.22 to −0.08]
Confidence: WLM	2.82 (1.36)	−0.23 [−0.3 to −0.16]	−0.14 [−0.21 to −0.07]	−.07 [−0.14 to 0.0]

Note. 99% confidence intervals. All scales from 1 to 6. All correlations Pearson’s *r*. *WL* weight loss, *WLM* weight loss maintenance

Participants with higher BMIs considered that weight loss and weight loss maintenance were more important than participants with lower BMIs did. However, participants with higher BMIs were also less confident in their ability to lose weight and to keep it off. BMI was not related to perceptions of past weight loss success, but higher BMI was linked to lower perceived weight loss maintenance success. Participants with higher numbers of previous lifetime weight loss attempts considered weight loss and weight loss maintenance to be more important than those with fewer weight loss attempts. In contrast, perceptions of success and confidence were negatively related to these same factors, such that participants with more numerous lifetime previous weight loss attempts felt less successful and confident at keeping weight off. Participants with greater numbers of health conditions felt less confident at losing weight.

### Motivating factors for weight loss

Participants were asked whether specific motivating factors prompted their decision to lose weight in their current or most recent attempt. Most participants reported that a wish to improve their overall health was a motivating factor (77%), followed by unhappiness with body shape/size (64%), unhappiness with appearance (61%), wishing to reduce health risks (60%), wishing to improve physical fitness (55%), and unhappiness with clothes fitting poorly (52%). Advice from a primary care professional (GP/nurse) was reported as a motivating factor by 30% of participants. Other motivating factors included being inspired by others who had recently lost weight (17%), wanting to lose weight for an upcoming event (16%), losing weight in response to comments from other people (14%), and advice from a non-primary care health professional (7%).

Participants who were motivated by primary care advice to lose weight tended to be older and have a greater number of health conditions (*r* = 0.12 and *r* = 0.13 respectively, both *p* < 0.001), but they did not tend to have a higher BMI (*r* = 0.08, *p* = 0.005). Instead, participants with a higher BMI were more likely to identify a health scare as a motivating factor (*r* = 0.12, *p* < 0.001). Younger participants and those with a higher BMI were more likely to be motivated by unhappiness with appearance (*r* = 0.11 and *r* = −0.27 respectively, both *p* < 0.001). Younger participants were also more likely to be motivated by unhappiness with body size/shape (*r* = −0.22, *p* < 0.001), wishing lose weight for a special event (*r* = −0.20, *p* < 0.001), poor fit of clothing (*r* = −0.14, *p* < 0.001) and wishing to improve fitness (*r* = −0.11, *p* < 0.001). Participants with higher numbers of previous weight loss attempts were more likely to cite unhappiness with appearance as a motivating factor (*r* = −0.12, *p* < 0.001) but were no more likely to endorse any of the other motivating factors.

### Weight loss strategies and self-weighing

**Fig. 1:**
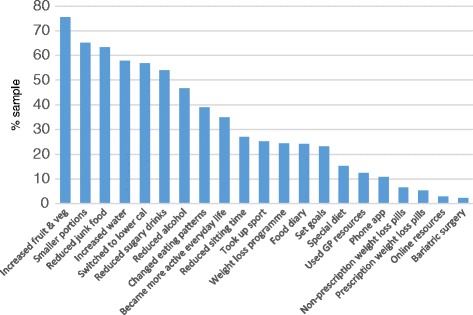
Proportion of participants who used specific weight loss strategies (*N* = 1309)

Participants were asked whether they had used specific weight loss strategies in the previous 12 months. Figure 1 shows the proportion of participants who reported using each strategy. Self-directed lifestyle modifications were most commonly used, including increasing fruit and vegetable and water consumption (75% and 58% respectively), switching to lower calorie food/drink items (57%), reducing intake of unhealthy foods and drinks (junk food: 63%; sugary drinks: 54%; alcohol: 47%), reducing portion sizes (65%), and changing eating patterns (e.g., timing of meals: 39%). Physical activity was used by a smaller proportion of patients: 37% became more active, 27% reduced sedentary time, and 26% took up a sport. Participants used strategies involving professional support less frequently: these included membership of a commercial or local authority weight-loss programme (25%), using prescription weight loss pills (i.e., Orlistat: 4%) or using resources from primary care (12%). Bariatric surgery was used by only 2%. A minority of participants used self-regulatory strategies, which included behavioural self-monitoring using a food diary or phone app (e.g., My Fitness Pal: 11%) and setting goals (23%). Participants who used each of these strategies were significantly younger than those who had not used them (all *p* values <0.001). Most participants weighed themselves at least weekly (weekly: 30%; several times per week: 8%; daily: 11%). The others weighed themselves every couple of weeks (12%), once a month (20%), or never (11%). The majority of respondents weighed themselves at home (73%) rather than in another setting.

### Professional weight loss support

A minority (40%) of the whole sample had sought some form of professional support in relation to their weight in the past 12 months: from a GP or practice nurse (20%), commercial weight loss consultant (13%), dietitian (9%) or exercise specialist (7%). Nutritionists, psychologists, and secondary care staff were rarely used for weight loss support (all <2%). Recent use of professional support was significantly higher amongst participants currently trying to lose weight (49%) than participants not currently trying (24%; χ^2^_(2)_ = 54.7, *p* < 0.001). Of the subgroup of participants trying to lose weight (*n* = 810), 25% had consulted a GP or practice nurse: these individuals were highly likely to use GP resources in their weight loss attempt (*r* = 0.47, *p* < 0.001) and likely to report that GP or practice nurse advice motivated their weight loss attempt (*r* = 0.17, *p* < 0.001).

### Physical activity

Levels of physical activity that met current recommendations of at least 75 min of vigorous activity per week or 150 min of moderate activity per week were reported by 42% of participants. A greater proportion of men (49%) than women (37%; *χ*^*2*^_(2)_ = 18.1, *p* < 0.001) did so. Participants with recommended levels of physical activity had a slightly lower BMI than those who did not, but this did not reach the threshold for statistical significance (*r* = 0.08 *p* < 0.01). The majority (60%) of participants reported no vigorous physical activity at all, whilst 55% reported no moderate-intensity physical activity, and 15% reported doing no walking each week. The mean reported time spent walking per day was 15 min (median = 1 h).

### Barriers to weight loss

Participants were asked whether they experienced specific barriers to weight loss during their most recent attempt. Commonly reported barriers included feeling tired (49%), stressed (41%), demotivated (38%) or low (37%). Participants reported feeling that they were missing out (30%), getting bored of routine (28%), did not have enough time (25%), and had competing priorities (17%). Thirty-seven percent of participants also reported that health conditions made it harder to lose weight: conditions perceived as barriers included arthritis (6% of total sample), diabetes (5%), joint/spinal problems (5%), thyroid problems (3%), cardiac problems (2%), and respiratory conditions (2%). Participants with greater numbers of barriers to weight loss had higher BMIs (*r* = 0.22, *p* < 0.001) and reported higher numbers of health conditions (*r* = 0.16, *p* < 0.001). They felt less confident about losing weight (*r* = −0.15, *p* < 0.001) and maintaining weight loss (*r* = −0.23, *p* < 0.001), and considered themselves less successful at weight loss maintenance (*r* = −0.17, *p* < 0.001).

### Free-text comments about weight control

At the end of the survey, participants were invited to provide any additional comments in a free-text box. Comments were provided by 498 (38%) participants. Using a content analytic approach, we double-coded participant comments for common themes. Most provided further details of barriers to weight loss, including physical health (*n* = 87), pain (*n* = 19), limited mobility (*n* = 19), occupational demands (*n* = 42), family commitments (*n* = 29), poor psychological health (*n* = 52), and medication causing weight gain (*n* = 11). Participants stated that insufficient help was available to help them lose weight (*n* = 23), and highlighted the particular difficulty of maintaining weight loss once it had been achieved (*n* = 27). A smaller number described successful weight loss, all with commercial providers (*n* = 15). Others reflected on experiences in primary care that influenced their efforts to lose weight loss (*n* = 20): most participants described their experiences negatively, highlighting an absence of encouragement or advice.

## Discussion

The results of this cross-sectional survey showed that primary care patients with objectively-recorded obesity were actively engaged in weight loss attempts, considered weight control to be important and had extensive previous experience of trying to lose weight and keep it off. However, participants’ confidence and perceived success were low for weight loss and still lower for weight loss maintenance. Weight loss attempts tended to be self-guided (unsupported), using strategies without clear evidence of effectiveness, and were impeded by both psychological and physical health-related barriers. Overall, responses suggested an unmet need for informational support (particularly for weight loss maintenance), sign-posting to structured services, and recognition of the complex experiential and motivational context in which patients attempt weight control.

The majority of survey participants were actively trying to lose weight, indicating that guidance on *how* to lose weight is likely to be more useful to patients than advice to simply initiate weight loss. It appears more fruitful to capitalise upon and shape patients’ existing efforts than to assume that they are currently disengaged. The proportion actively trying to lose weight (62%) resembled previous studies with participants with overweight and obesity [21–24] but was lower than in HSE participants with obesity (76%; [12]). Amongst those trying to lose weight, a minority had recently sought professional weight loss support and only a quarter had sought advice from primary care in the past 12 months, despite health concerns being the most common motivating factor for weight loss, in keeping with previous research [24, 25]. Those that did seek primary care advice were highly likely to go on to use primary care resources for weight loss and cite the advice received as a motivating factor in their weight loss attempt. Overall, this indicates that primary care advice and signposting has the potential to augment the activities of primary care patients not already using it, and complements existing evidence that such support is acceptable to, and would be viewed favourably by, patients [26, 27].

The most commonly-used weight loss strategies were those with inconclusive evidence for weight loss effectiveness, so providing information about the best-evidenced strategies may be useful to many primary care patients with obesity. Popular strategies were coherent with self-guided ‘healthy lifestyle’ advice but have not been shown to be effective weight loss interventions in isolation: these included increasing fruit and vegetable consumption [28, 29], reducing portion size [30] and drinking more water [31]. Strategies with stronger evidence bases, such as following a structured weight loss programme [32], using Orlistat [33], and goal setting and self-monitoring [34, 35] were used less frequently. Indeed, the finding that most participants’ weight loss strategies were essentially self-guided (unsupported) is of particular concern given participants’ limited confidence for weight loss and weight loss maintenance. Although we did not ask about participants’ weight loss maintenance strategies, it is notable that overall confidence for maintenance was lower than that for weight loss, suggesting a greater need for support in this area. Overall, information that explicitly differentiates ‘health’ from ‘weight loss’ messages may be of particular benefit, as may recommendations for existing, supported weight loss programmes or drug regimens [32, 33].

Participants were less confident about maintaining weight loss than losing weight, and felt less successful at maintenance than loss, indicating the need for maintenance-specific advice and support. Whilst effective weight loss maintenance interventions exist [36], scalable interventions do not yet [37], and thus maintenance provision is limited in both commercial and local authority contexts. UK clinical guidelines contain minimal maintenance-specific content [5–7]. Maintenance confidence and success were lowest amongst those with higher BMIs and more previous weight loss attempts, possibly because repeated attempts and increasing weight ultimately reduced participants’ belief that they are capable of keeping weight off over the longer term [38]. Similar relationships were not seen with confidence and success for weight loss. Overall, these findings highlight the importance of sign-posting patients to support, even if they have already achieved some weight loss independently, and of prompting patients to plan ahead for maintenance in discussions of weight loss strategies.

Participants at greatest apparent clinical risk, i.e., those with the highest BMIs and most numerous health conditions, were also those with the lowest confidence for weight loss and weight loss maintenance, indicating that this group of individuals may be in particular need of support. All the aforementioned factors have been shown to be prognostic of poor weight loss and weight loss maintenance in previous research [39, 40]. This group of participants also reported more numerous previous weight loss attempts and barriers, and lower perceived weight loss maintenance success. Health conditions were a key barrier to weight loss for participants, particularly those who were older, had higher BMIs, and lower weight loss confidence. However, this group still considered weight loss and weight loss maintenance to be important and were more likely to seek primary care support than others. These findings underscore the need to proactively discuss weight loss with patients with high BMI and multimorbidity, even though primary care staff may avoid doing so when they perceive there are other priorities such as health conditions [41].

Concerns about bodily appearance, size, and shape were particularly pertinent motivating factors to younger patients, as was the wish to become fitter, whereas health concerns were reported as motivating factors by patients of all ages. The latter finding is inconsistent with prior research indicating that health-related motivation for weight loss increases with age, particularly in men [42], but it may be attributable to the comparatively higher mean participant age in the current study. The former finding suggests that primary care staff should be particularly sensitive to the importance of body image and drive to improve fitness amongst younger patients. Counter-intuitively, but in keeping with recent meta-analytic findings [24], younger participants had higher numbers of previous weight loss attempts than older participants, potentially a consequence of greater social pressure to lose weight in this group [43]. Such pressure may also explain our finding that those with greater numbers of weight loss attempts were more likely to be motivated by appearance concerns.

A key strength of the study was the recruitment of participants with recently-recorded objective obesity from systematic searches of GP lists. Weight studies reliant upon self-selection tend to disproportionately attract women [44], whereas men and women were more equally represented in the current sample. Surveys using self-reported BMI data as a determinant of eligibility are vulnerable to the effects of systematic underreporting of weight and over-reporting of height [45]. Here, eligibility was determined using objective data from practice records. The aforementioned underreporting of current weight and height may explain the finding that, according to the self-report survey data, only 83% of the sample was classified as obese, with 17% reporting a BMI in the overweight range, and 0.5% in the healthy weight range. Alternatively, inaccuracies in patient records may explain this, as found in a previous study [46]; a consequent study limitation is that patients with a BMI in the eligible range may have been missed because their weight status has not been correctly coded. The response rate achieved (23%) is comparable to that of similar ad hoc surveys asking about health and lifestyle behaviours [47–49], but lower than other, larger surveys such as the GP Patient Survey [50]. More than 90% of participants in the current study self-identified as white. This limits the extent to which the study findings are generalizable to more diverse populations, although it accurately reflects the ethnic composition of the geographical area in which the survey was distributed [51]. The data presented are cross-sectional and, as such, causal inferences are precluded. Finally, data were not available regarding individual participants’ frequency of primary care usage, and the sample’s demographic composition (e.g., approximately equal numbers of men and women) differs from the typical composition of patients utilising UK primary care consultations [52] although the mean participant age is broadly consistent with it.

## Conclusion

We examined the weight loss support needs of GP patients with objectively-recorded obesity in North East England, and identified the need for informational, structural, and weight loss maintenance-specific support. Study participants were motivated to lose weight and keep it off, but lacked the confidence and understanding of effective strategies required to do this. A minority had sought structured weight management support and fewer still advice from primary care, but those that did found it motivating and useful. GP provision of demonstrably-effective brief referral interventions to structured weight loss support [8] may be acceptable to these patients, to augment their current weight management activities and to optimise limited GP time and resources.

## Additional file

Additional file 1: Study questionnaire. Questionnaire used in study. (PDF 462 kb)

## Abbreviations

BMI: Body mass index
GP: General practitioner
