# Supplementary material for: Support needs of patients with obesity in primary care: a practice-list survey
Source: BMC Fam Pract. 2018 Jan 8;19:6. doi: 10.1186/s12875-017-0703-4 (PMC5759249; doi:10.1186/s12875-017-0703-4)
Supplement: Additional file 1: — Study questionnaire. Questionnaire used in study. (PDF 462 kb) [file 12875_2017_703_MOESM1_ESM.pdf]

Institute of Health&Society

Newcastle University

QUESTIONNAIRE PRIVATE & CONFIDENTIAL

Thank you for taking the time to fill out this questionnaire

SECTION A: WEIGHT HISTORY

This section asks about your weight and any weight changes you have experienced.

A1. What do you currently weigh?

__________ stones __________ pounds *OR* __________ kilograms

A2. How tall are you?

__________ feet __________ inches *OR* __________ meters __________ centimeters

A3. What is *the most* you have ever weighed since reaching your current height?
(not including pregnancy, if applicable)

________ stones ________ pounds *OR* ________ kilograms

A4. What is *the least* you have ever weighed since reaching your current height?
(excluding any weight loss due to illness)

__________ stones __________ pounds *OR* __________ kilograms

__________ stones __________ pounds *OR* __________ kilograms

A5. For how long have you been at your current weight or close to it?
(within 4lbs / 2kg)

__________ years __________ months __________ weeks __________ days

A6. Which of these statements best describes your weight over the last 12 months?
*(Please tick **ONE** only)*

My weight has …

☐ stayed about the same ☐ gone up

☐ gone down ☐ gone up and down

SECTION B: WEIGHT MANAGEMENT

This section asks about any efforts you have made to lose weight or keep weight off once you've lost it.

B1. When was the last time that you tried to lose weight? *(Please tick **ONE**)*

- ☐ I am trying to lose weight at the moment
- ☐ In the last 3 months *(skip to B2)*
- ☐ In the last 6 months *(skip to B2)*
- ☐ In the last 12 months *(skip to B2)*
- ☐ More than 12 months ago *(skip to B2)*
- ☐ Never **(skip to SECTION C)**

B1b. For how long has your current attempt to lose weight lasted so far?
(Please specify as appropriate)

__________ years __________ months __________ weeks __________ days ☐ n/a

B2. How many times in your life have you intentionally lost the <u>total</u> amount of weight listed?
*(Please respond to **EVERY ITEM**).*

1-4 pounds (0.5-2kg)	__________ times	☐ n/a
5-10 pounds (2-5kg)	__________ times	☐ n/a
11-20 pounds (6-9kg)	__________ times	☐ n/a
21 pounds (10kg) or more	__________ times	☐ n/a

Thinking about your previous weight management efforts *overall* …

B3. How *<u>successful</u>* have you been in your efforts to…
(Please circle a number on each line)

	Not successful ←				→ Very successful		
**Lose weight**:	1	2	3	4	5	6	☐ n/a
**Keep weight off**: *(once you've lost it)*	1	2	3	4	5	6	☐ n/a

B4. How *<u>confident</u>* are you in your ability to…
(Please circle a number on each line)

	Not confident ←				→ Very confident		
**Lose weight**:	1	2	3	4	5	6	☐ n/a
**Keep weight off**: *(once you've lost it)*	1	2	3	4	5	6	☐ n/a

B5. How *important* is it for you to…
(Please circle a number on each line)

	Not important ←				→ Very important		
**Lose weight**:	1	2	3	4	5	6	☐ n/a
**Keep weight off**: *(once you've lost it)*	1	2	3	4	5	6	☐ n/a

B6. Thinking about your weight management efforts in the last 12 months (including any current attempts), what did you *do* to manage your weight?
*(Please tick **ALL** that apply)*

☐ Ate smaller portions ☐ n/a
☐ Set yourself weight management goals
☐ Switched to food with fewer calories
☐ Kept a food diary
☐ Ate more fruit, vegetables and/or salads
☐ Drank more water
☐ Changed eating patterns and/or times (e.g. didn't eat late at night)
☐ Reduced/avoided alcohol
☐ Reduced/avoided sugary drinks
☐ Ate less junk food (e.g. takeaways)
☐ Took up a sport or hobby involving physical activity
☐ Became more active in everyday life (e.g. walking to work)
☐ Spent less time sitting down
☐ Used resources or services provided by your GP practice
☐ Took non-prescription weight loss pills/supplements (*specify*) ____________________
____
☐ Bariatric surgery (e.g. gastric band) (*specify*) ___________________________________
☐ Joined a weight loss programme (*specify*) _____________________________________
____
☐ Used online resources e.g. NHS choices (*specify*) ______________________________
____
☐ Used a phone app (*specify*) ____

____

☐ Followed a special diet e.g. eliminated food groups (*specify*) ____________________

____

☐ Took prescribed weight loss medication (*specify*) ______________________________

____

☐ Other *(specify)* ____

____

B7. Thinking about your <u>most recent</u> weight management efforts (including any current efforts) what *<u>gets in the way of</u>* managing your weight?
*(Please tick **ALL** that apply)*

☐ Feeling stressed ☐ n/a
☐ Feeling tired
☐ Not having time
☐ Feeling low
☐ Feeling demotivated
☐ Feeling bored of routine
☐ Feeling like you are 'missing out' (e.g. food you enjoy)
☐ Having other competing priorities
☐ Health condition (*specify*) ____
☐ Other (*specify*) ____

B8. Thinking about your <u>most recent</u> weight management efforts (including any current efforts) what *<u>motivated</u>* you to manage your weight?
*(Please tick **ALL** that apply)*

☐ A GP or practice nurse advised me to lose weight ☐ n/a
☐ I had a health scare (e.g. heart attack, diagnosis)
☐ A health professional (NOT GP or practice nurse) advised me to lose weight
☐ I could no longer fit into clothes I wanted to wear
☐ I was inspired by family/friend(s)/other people who lost weight

- ☐ I wanted to lose weight for an event (e.g. wedding, milestone birthday)
- ☐ I wanted to improve my overall health
- ☐ I wanted to improve my physical fitness
- ☐ I wanted to reduce my risk of suffering poor health in the future
- ☐ Family/friends commented on my weight
- ☐ I was unhappy with my general appearance
- ☐ I was unhappy with my body shape and size
- ☐ I reached a life-time high weight
- ☐ Other (please write) ____

____

B9. In the past 12 months, have you sought help from any of the following people to lose weight? *(Please tick **ALL** that apply)*

- ☐ GP/practice nurse
- ☐ Exercise specialist/trainer
- ☐ Psychologist
- ☐ Dietician
- ☐ Nutritionist
- ☐ Hospital consultant
- ☐ Commercial weight loss consultant/ trainer
- ☐ Other ____

B10. How often do you weigh yourself? *(Please tick **ONE**)*

- ☐ Never
- ☐ Once per month or less
- ☐ Every couple of weeks
- ☐ Once per week
- ☐ Several times per week
- ☐ Daily
- ☐ Other *(please write how often)*

____

B11. Where do you weigh yourself or get weighed? *(Please tick **ALL** that apply)*

- ☐ I **do not** weigh myself/get weighed
- ☐ At home
- ☐ At the GP practice/hospital/clinic
- ☐ At the gym/exercise class
- ☐ At a weight-loss club
- ☐ Other *(please write where)*

____

B12. Which kind of scales do you use? *(Please tick **ALL** that apply)*

- ☐ None
- ☐ Dial scale
- ☐ Digital scale
- ☐ Wifi scale
- ☐ Other *(Please write)*

____

SECTION C: PHYSICAL ACTIVITY (IPAQ-SF)

Think about all the **vigorous** activities that you did in the **last 7 days**. **Vigorous** physical activities refer to activities that take hard physical effort and make you breathe much harder than normal. Think *only* about those physical activities that you did for at least 10 minutes at a time.

C1. During the last 7 days, on how many days did you do vigorous physical activities like heavy lifting, digging, aerobics, or fast bicycling? *(Tick ALL that apply)*

__________ days per week ☐ No vigorous physical activities *(Skip to C3)*

C2. How much time did you usually spend doing vigorous physical activities on one of those days?

__________ hours per day __________minutes per day ☐ Don't know/Not sure

Think about all the **moderate** activities that you did in the **last 7 days**. **Moderate** activities refer to activities that take moderate physical effort and make you breathe somewhat harder than normal. Think only about those physical activities that you did for at least 10 minutes at a time.

C3. During the last 7 days, on how many days did you do moderate physical activities like carrying light loads, bicycling at a regular pace, or doubles tennis? Do not include walking.

__________ days per week ☐ No moderate physical activities *(Skip to C5)*

C4. How much time did you usually spend doing moderate physical activities on one of those days?

__________ hours per day __________ minutes per day ☐ Don't know/Not sure

Think about the time you spent **walking** in the **last 7 days**. This includes at work and at home, walking to travel from place to place, and any other walking that you might do solely for recreation, sport, exercise, or leisure.

C5. During the last 7 days, on how many days did you walk for at least 10 minutes at a time?

__________ days per week ☐ No walking *(Skip to C7)*

C6. How much time did you usually spend walking on one of those days?

__________ hours per day __________ minutes per day ☐ Don't know/Not sure

The next question is about the time you spent **sitting** on weekdays during the **last 7 days**. Include time spent at work, at home, while doing course work and during leisure time. This may include time spent sitting at a desk, visiting friends, reading, or sitting or lying down to watch television.

C7. During the last 7 days, how much time did you spend sitting on a week day?

__________ hours per day __________ minutes per day ☐ Don't know/Not sure

SECTION D: ABOUT YOU

This section asks for some details about you.

D1. Are you male or female? ☐ Male ☐ Female ☐ I prefer not to say

D2. How old were you on your last birthday? ______________ years

D3. What is the full postcode where you currently live? *(Please write both parts of your postcode in full in the boxes below)*

☐ ☐ ☐ ☐ ☐ ☐ ☐ ☐

D4. Which of the following qualifications do you have? *(Please tick **ALL** that apply)*

☐ 1 or more O levels, CSEs, GCSEs or equivalents
☐ 1 or more A levels or equivalents
☐ Vocational qualifications such as NVQ, GNVQ, or City & Guilds
☐ A degree such as BA or BSc, MA or MSc
☐ No formal qualifications
☐ Prefer not to say
☐ Other *(please write)* ____

D5. What is your current marital status? *(Please tick **ONE)***

☐ Single ☐ Separated ☐ I prefer not to say
☐ Cohabiting ☐ Divorced
☐ Married (including civil partnership) ☐ Widowed

D6. How would you describe your ethnicity?

____ ☐ I prefer not to say

D7. Do you consider yourself to have any of the following conditions?
*(Please tick **ALL** that apply)*

- ☐ Diabetes
- ☐ Pain/discomfort
- ☐ Mobility problems
- ☐ Anxiety/depression
- ☐ High blood pressure
- ☐ None
- ☐ Other (*specify*) ____
- ☐ I prefer not to say

D8. Do you have any disability or health problem that makes it difficult for you to …
*(Please tick **ALL** that apply)*

☐ Go out on foot ☐ Get in or out of a car ☐ Use public transport

D9. In general, would you say your health is… *(please tick **ONE**)*

☐ Excellent ☐ Very good ☐ Good ☐ Fair ☐ Poor

☐ I prefer not to say

If you have any additional comments you would like to make, please write them here:

Thank you very much for completing this questionnaire.

That is the end of the questionnaire. Please check that you have answered all questions on each page. Please then return the questionnaire in the envelope provided. If you have any questions or concerns about the questionnaire or research, please contact a member of the study team:

Dr Elizabeth Evans
Phone: 0191 208 5591
Email: Elizabeth.evans@newcastle.ac.uk

Alex Smith
Phone: 0191 208 7394
Email: Alexandra.smith@newcastle.ac.uk
